# The Effects of Insulin Resistance on Individual Tissues: An Application of a Mathematical Model of Metabolism in Humans

**DOI:** 10.1007/s11538-016-0181-1

**Published:** 2016-06-15

**Authors:** Taliesin Pearson, Jonathan A. D. Wattis, John R. King, Ian A. MacDonald, Dawn J. Mazzatti

**Affiliations:** Centre for Mathematical Medicine and Biology, School of Mathematical Sciences, University of Nottingham, Nottingham, NG7 2RD UK; Queen’s Medical Centre, University of Nottingham Medical School, Nottingham, NG7 2UH UK; Unilever Research, 55 Merritt Blvd, Trumbull, CT USA

**Keywords:** Multicompartmental modelling, Metabolic flexibility, Insulin resistance

## Abstract

Whilst the human body expends energy constantly, the human diet consists of a mix of carbohydrates and fats delivered in a discontinuous manner. To deal with this sporadic supply of energy, there are transport, storage and utilisation mechanisms, for both carbohydrates and fats, around all tissues of the body. Insulin-resistant states such as type 2 diabetes and obesity are characterised by reduced efficiency of these mechanisms. Exactly how these insulin-resistant states develop, for example whether there is an order in which tissues become insulin resistant, is an active area of research with the hope of gaining a better overall understanding of insulin resistance. In this paper, we use a previously derived system of 12 first-order coupled differential equations that describe the transport between, and storage in, different tissues of the human body. We briefly revisit the derivation of the model before parametrising the model to account for insulin resistance. We then solve the model numerically, separately simulating each individual tissue as insulin resistant, and discuss and compare these results, drawing three main conclusions. The implications of these results are in accordance with biological intuition. First, insulin resistance in a tissue creates a knock-on effect on the other tissues in the body, whereby they attempt to compensate for the reduced efficiency of the insulin-resistant tissue. Second, insulin resistance causes a fatty liver, and the insulin resistance of tissues other than the liver can cause fat to accumulate in the liver. Finally, although insulin resistance in individual tissues can cause slightly reduced skeletal muscle metabolic flexibility, it is when the whole body is insulin resistant that the biggest effect on skeletal muscle flexibility is seen.

## Introduction

Whilst the human body expends energy constantly, the human diet consists of a mix of carbohydrates and fats delivered in a *discontinuous* manner. To deal with this sporadic supply of energy, there are transport, storage and utilisation mechanisms, for both carbohydrates and fats, around various tissues in the body.

The skeletal muscle of healthy lean subjects is able to switch its fuel source between mainly carbohydrates in the insulin-stimulated postprandial state to predominantly fats in the fasted state. The main regulatory hormone involved in metabolism is insulin, and hence, insulin-resistant states such as obesity and type 2 diabetes are characterised by a reduction in metabolic flexibility (Kelley and Mandarino 2000; Kelley 2005).

There are three broad categories of insulin-resistance or insulin-deficient states: (i) abnormal $\beta$-cell secretory product, which results in reduced insulin production in the pancreas; (ii) insulin antagonists in the blood plasma, due either to counterregulatory hormones or to non-hormonal bodies that affect insulin receptors or alter insulin-signalling effectiveness; (iii) the target tissue being defective in insulin action, due to defects either in the insulin receptors or to the effector systems. The third of these is of most interest here, since this form of insulin resistance is common in some obese and all type 2 diabetic subjects, although early-phase insulin secretion is also inhibited in some type 2 diabetic subjects (Olefsky 1981; Mizuno et al. 2007). Both obese and diabetic subjects exhibit lower plasma glucose disposal rates and increased basal glucose levels (Olefsky 1981; Mizuno et al. 2007; Prager et al. 1986).

In type 2 diabetes these effects are partly explained by impaired hepatic glucose uptake (Mizuno et al. 2007; Iozzo et al. 2003) and a reduction in the suppression of hepatic glucose output by insulin (Kotronen et al. 2008). Also, skeletal muscle glucose uptake is impaired in type 2 diabetes (Phielix and Mensink 2008). In non-diabetic obese subjects, there is reduced skeletal muscle glucose uptake (den Boer et al. 2006) and reduced hepatic glucose output suppression from insulin (Olefsky 1981; Prager et al. 1986). Furthermore, insulin-resistant subjects may experience increased hepatic lipogenesis, leading to larger changes in hepatic triglyceride concentrations in the postprandial state, a counterintuitive result since it is an insulin-stimulated pathway. The reason for this effect is that the blood supply from the pancreas goes directly to the liver, and the initial response to insulin resistance is increased insulin secretion. However, Peterson et al. (2007) have shown that insulin-resistant subjects have a significantly larger reduction in muscle glucose uptake, indicating that insulin resistance affects different tissues to different degrees (Peterson et al. 2007), the liver being less severely affected, as noted by Bock et al. (2007).

Not only does insulin resistance affect glucose metabolism, it also affects adipose tissue, which exhibits a reduced insulin suppression of free fatty acid release (den Boer et al. 2006). Insulin-resistant subjects also suffer from reduced clearance of adipose tissue triglyceride and lower skeletal muscle triglyceride clearance (Bickerton et al. 2008). Currently, fatty liver disease is a major area of interest as it relates to insulin resistance and obesity. It is not currently known whether obesity causes or is caused by fatty liver disease or whether both are the cause of some other process (Rijkelijkhuizen et al. 2009). It has been shown that postprandial hepatic triglyceride release is higher in people with fatty liver disease than healthy people, although the basal secretion rate is the same. This result implies that it is not a higher fat presence in the liver that causes the increased output, but rather insulin resistance (Adiels et al. 2007).

In this paper, we use a mathematical model which was derived in detail in a previous paper Pearson et al. (2014) to simulate insulin resistance. In Sect. 2.1, we briefly recap the model, and in “Appendix 1”, we use experimental data available from the literature to parametrise insulin resistance in various tissues of the body.

The model neglects protein metabolism, since those processes are largely separate, and would vastly complicate the model were they to be included. Carbohydrate and fat metabolism have more significant interactions and can be modelled in a useful way relatively simply. The results of simulations of such models aid our understanding of metabolism in the body as a whole. Our models are kept as simple as possible by neglecting another feature present in the early onset of insulin resistance: namely, that the pancreas will supply higher levels of insulin to counterbalance small and moderate levels of insulin resistance in other tissues. Our first aim is to isolate the effects of insulin resistance in other tissues. In the later works Pratt et al. (2015b), Pratt (2015), we plan to include terms to model the glucose sensitivity of insulin production by the pancreas.

The remainder of Sect. 2 discusses each tissue in turn, and its response to glucose, fatty acids and TAG. In Sect. 3, we compare the results of simulations of insulin-resistant subjects and healthy subjects. We first simulate the case of each individual tissue in our model (liver, skeletal muscle and adipose tissue) being insulin resistant whilst the other tissues are normal; later, we consider whole-body insulin resistance. Our motivation for this approach is to investigate the effect of insulin resistance of an individual tissue on whole-body metabolism, in order to provide some insight into the order in which tissues may become insulin resistant in the development of type 2 diabetes. In Sect. 4, we summarise our results and draw conclusions, as well as discuss possible directions for future work.

## Mathematical Model

### Preliminaries

**Fig. 1:**
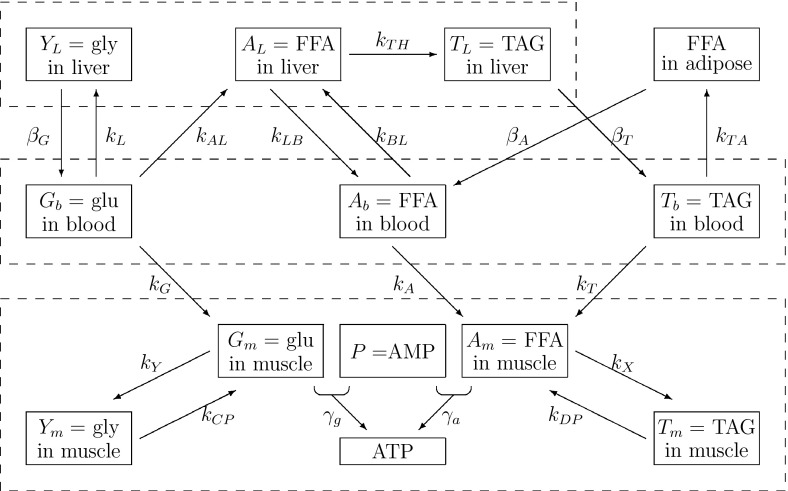
Diagram of the biochemical reaction network used in this paper. *Dashed boxes* indicate the different compartments of the model with the liver at the *top*, the blood plasma in the *middle* and skeletal muscle at the *bottom*. *glu* glucose, *gly* glycogen

The mathematical model we use in this paper was derived in detail in an earlier paper (Pearson et al. 2014). In this section, we briefly recap the model whilst generalising it to encompass insulin resistance. The model is multicompartmental in nature and describes the concentrations of glucose, fatty acids, TAG, glycogen and insulin in the various main tissues of the human body involved in metabolism. We include skeletal muscle, the liver, adipose tissue [as a supply of free fatty acids (FFAs) and sink for triglycerides (TAGs)] and the blood plasma, which acts as a transportation medium between the tissues. The relevant metabolic pathways are shown in Fig. 1.

As indicated in Fig. 1, the pathways we model are: the uptake of glucose from the blood into the liver with rate $k_L$ (and release back into the blood with rate $\beta _G$) and skeletal muscle ($k_G$), the uptake of TAG from the blood into adipose tissue ($k_{TA}$) and skeletal muscle ($k_T$) as well as the release into the blood from the liver ($\beta _T$); and the uptake of FFA from the blood into the liver ($k_{BL}$) and skeletal muscle ($k_A$), as well as release into the blood from the liver ($k_{LB}$) and adipose tissue ($\beta _A$). In addition to these transport pathways via the blood plasma, we model some of the metabolic pathways within the liver and skeletal muscle. In the liver, some glucose is converted to glycogen for storage ($k_L$) and later release ($\beta _G$); in addition, some glucose is converted into FFA via glycolytic and lipogenesis pathways ($k_{AL}$)—this hepatic FFA can be released back into the plasma ($k_{LB}$) or converted into TAG in the liver ($k_{TH}$) to be stored and released later when the body needs it ($\beta _T$).

When TAG from the plasma is taken up into either adipose tissue or skeletal muscle, it is first broken down into FFA on the cell walls by the enzyme lipoprotein lipase. Hence, the uptake of both FFA and TAG from the plasma contributes to skeletal muscle FFA. The FFA in skeletal muscle can either be stored as TAG in the muscle or oxidised to form adenosine triphosphate (ATP). Similarly to the uptake of fats, glucose is also taken up into skeletal muscle, where it can either be stored as glycogen or oxidised to form ATP.

**Table 1:** Dimensional variables with their descriptions

Variable	Concentration of:
*I*	Plasma insulin
$G_b$	Plasma glucose
$T_b$	Plasma TAG
$A_b$	Plasma FFA
$Y_L$	Hepatic glycogen
$A_L$	Hepatic FFA
$T_L$	Hepatic TAG
*P*	Skeletal muscle AMP
$G_m$	Skeletal muscle glucose
$Y_m$	Skeletal muscle glycogen
$A_m$	Skeletal muscle FFA
$T_m$	Skeletal muscle TAG

In addition to the pathways shown in Fig. 1 the model also includes source terms in the form of time-dependent functions modelling input of glucose, FFA and TAG from the diet into the blood, via the terms $F_G,F_T$, and sink terms including the use of substrates from the plasma by the rest of the body, $S_G$, and the oxidation of FFA in the liver for energy, $S_L$.

**Table 2:** List of dimensional parameters together with brief description of each

Parameter	Description	Value
$M_G$	Glucose oxidation rate	
$M_A$	FFA oxidation rate	
$k_Y$	Glycogen synthesis rate (basal)	
$k_{YI}$	Glycogen synthesis rate (insulin stimulated)	
$k_{YP}$	Glycogen synthesis rate (AMP inhibited)	
$k_T$	Muscle triglyceride uptake rate (basal)	$2\times 10^{-4}$ mmol/l/min
$k_G$	Muscle glucose uptake rate (basal)	$3.6\times 10^{-3}$ mmol/l/min
$k_{GI}$	Muscle glucose uptake rate (insulin stimulated)	$1.75\times 10^7$ l/mmol
$k_{CP}$	Glycogenolysis rate (AMP stimulated)	
$k_{CI}$	Glycogenolysis rate (insulin inhibited)	0
$S_G$	Body glucose consumption rate	
$\mu$	AMP (P) creation rate	
$\lambda _\mathrm{I}$	Insulin degradation rate	
$k_1$	Insulin production rate	$6.97 \times 10^{-5}$ mmol/min
$k_2$	Insulin production rate	$8.36 \times 10^{-5}$ mmol / min
$k_{IG}$	Insulin production rate	
$k_{I2}$	Insulin production rate	
$k_{IA}$	Insulin production rate	
$k_A$	Muscle FFA uptake rate constant	
$\beta _A$	Basal FFA production rate	0.011 mmol/l/min
$\beta _T$	Basal triglyceride production rate	$2.25\times 10^{-4}$ mmol/l/min
$\beta _G$	Basal glucose production rate	0.019 mmol/l/min
$\alpha$	Plasma volume/skeletal muscle volume	0.17
$\gamma _a$	Number of P molecules used in FFA oxidation	
$\gamma _g$	Number of P molecules used in glucose oxidation	
$\lambda _P$	Degradation of P in the absence of any other process	
$k_{GL}$	Insulin inhibition rate of glucose from liver	$1.06 \times 10^{14}$ l$^2$/pmol$^2$
$k_{TL}$	Insulin inhibition rate of TAG from liver	$2.5 \times 10^{6}$ l/mmol
$k_{AA}$	Insulin inhibition rate of FFA from adipose tissue	$2 \times 10^{14}$ l$^2$/pmol$^2$
$k_{TA}$	Adipose TAG uptake const (basal)	$0.5 \times 10^{-4}$ min$^{-1}$
$k_{AI}$	Adipose TAG uptake const (insulin stimulated)	$5 \times 10^6$ l/mmol
$k_X$	Muscle TAG synthesis rate const (basal)	
$k_{XI}$	Muscle TAG synthesis const (insulin stimulated)	0
$k_{XP}$	Muscle TAG synthesis const (AMP inhibited)	0
$k_{DP}$	Muscle TAG usage const (AMP stimulated)	
$k_{DI}$	Muscle TAG usage const (insulin inhibited)	0
$k_L$	Liver glucose uptake rate	$2 \times 10^4$ l/mmol/min
$Y_\mathrm{max}$	Maximum potential glucose stored in liver	310 mmol/l
$\eta$	Liver volume/skeletal muscle volume	0.064
$k_{AL}$	Rate of conversion of glucose to FFA (glycolysis)	
$k_{AS}$	Insulin inhibition rate of hepatic FFA oxidation	
$k_{BL}$	Rate of uptake of plasma FFA into the liver	
$k_{LB}$	Rate of release of hepatic FFA into blood plasma	
$k_{TH}$	Rate of conversion of FFA to TAG in the liver	
$S_L$	Rate of oxidation of FFA in the liver	
$B_T$	Delay from feeding to triglyceride reaching blood	
$B_G$	Delay from feeding to glucose reaching blood	
$k_{FG}$	Rate of uptake of glucose to blood	
$k_{FT}$	Rate of uptake of triglyceride to blood	
$\theta _G$	Proportion of carbohydrates in diet	
$\theta _T$	Proportion of triglyceride in diet	
*F*	Total calorific content of diet	

Values of zero correspond to processes which are straightforward to include in the model, but are less significant and harder to parametrise, so for simplicity, we set their values to zero

We model the concentration of insulin in the plasma by assuming a production term with nonlinear dependence on glucose given by $f_0(G_b)$, see (2), and a smaller linear dependence on FFA in the plasma, with constant of proportionality $k_{IA}$. This latter term ($k_{IA}A_b$) is small in comparison with the glucose-dependent term ($f_0$), and in our simulations meals are combinations of carbohydrates and fatty acids. The model is described by the variables listed in Table 1, and the rate parameters are summarised in Table 2, the kinetics being expressed in the following equations that comprise the dimensionless model (see Pearson et al. 2014 for the non-dimensionalisation):

1 $$\begin{aligned} \frac{\mathrm{d}I}{\mathrm{d}t}= & {} f_0(G_b) + k_{IA} A_b - \lambda _I I, \end{aligned}$$

2 $$\begin{aligned} f_0(G_b)= & {} k_1 + k_2 \mathrm{erf}( (G_b -v)/c) , \end{aligned}$$

3 $$\begin{aligned} \alpha \frac{\mathrm{d}G_b}{\mathrm{d}t}= & {} \left( \frac{\beta _G}{1+\sigma _L k_{GL} I^2} \right) f_1(Y_L) - S_G G_b \nonumber \\&- k_G(1+\sigma _G k_{GI}I) G_b - k_L \sigma _Y I G_b f_2(Y_L) - k_{AL} I G_b + F_G(t) , \end{aligned}$$

4 $$\begin{aligned} \alpha \frac{\mathrm{d}T_b}{\mathrm{d}t}= & {} \left( \frac{\beta _T}{1+\sigma _T k_{TL} I} \right) f_3(T_L) - k_TT_b - \phi _{AT} k_{TA}(1+\sigma _{AT} k_{AI} I ) T_b + F_T(t) , \end{aligned}$$

5 $$\begin{aligned} \alpha \frac{\mathrm{d}A_b}{\mathrm{d}t}= & {} \frac{\phi _A \beta _A}{1+\sigma _A k_{AA}I^2} - k_AA_b - k_{BL}A_b + k_{LB}A_L , \end{aligned}$$

6 $$\begin{aligned} \eta \frac{\mathrm{d}Y_L}{\mathrm{d}t}= & {} k_L \sigma _Y I G_b f_2(Y_L) -\left( \frac{\beta _G}{1+\sigma _L k_{GL}I^2}\right) f_1(Y_L) , \end{aligned}$$

7 $$\begin{aligned} \eta \frac{\mathrm{d}A_L}{\mathrm{d}t}= & {} k_{AL}IG_b-\frac{S_LA_L}{1+k_{AS}I} + k_{BL}A_b-k_{LB}A_L - k_{TH}IA_L , \end{aligned}$$

8 $$\begin{aligned} \eta \frac{\mathrm{d}T_L}{\mathrm{d}t}= & {} k_{TH}IA_L- \left( \frac{\beta _T}{1 + \sigma _T k_{TL} I } \right) f_3(T_L) , \end{aligned}$$

9 $$\begin{aligned} \frac{\mathrm{d}P}{\mathrm{d}t}= & {} \mu - {\lambda }_P P - {\gamma }_a M_A A_m P - \sigma _G {\gamma }_g M_G P I G_m , \end{aligned}$$

10 $$\begin{aligned} \frac{\mathrm{d}G_m}{\mathrm{d}t}= & {} k_G(1+\sigma _G k_{GI}I)G_b - \sigma _G M_G P I G_m - k_Y \left( \frac{1+\sigma _Gk_{YI}I}{1+k_{YP}P} \right) G_m\nonumber \\&+ \frac{k_{CP} P Y_m}{1+\sigma _Gk_{CI}I}, \end{aligned}$$

11 $$\begin{aligned} \frac{\mathrm{d}Y_m}{\mathrm{d}t}= & {} k_Y \left( \frac{1+\sigma _Gk_{YI}I}{1+k_{YP}P} \right) G_m - \frac{k_{CP} P Y_m}{1+\sigma _Gk_{CI}I}, \end{aligned}$$

12 $$\begin{aligned} \frac{\mathrm{d}A_m}{\mathrm{d}t}= & {} k_TT_b +k_AA_b - M_A P A_m - k_X \left( \frac{1+k_{XI}I}{1+k_{XP}P} \right) A_m + \frac{k_{DP} P T_m}{1+k_{DI}I} ,\qquad \end{aligned}$$

13 $$\begin{aligned} \frac{\mathrm{d}T_m}{\mathrm{d}t}= & {} k_X \left( \frac{1+k_{XI}I}{1+k_{XP}P} \right) A_m - \frac{k_{DP} P T_m}{1+k_{DI}I} . \end{aligned}$$

The source terms $F_G(t)$ and $F_T(t)$ are given by

14 $$\begin{aligned}&F_G(t) = \displaystyle \frac{F\theta _Gt}{B_G^2}\mathrm{e}^{-t^2/2B_G^2} , \qquad F_T(t) = \displaystyle \frac{F\theta _Tt}{B_T^2}\mathrm{e}^{-t^2/2B_T^2} ,&\end{aligned}$$

respectively, representing glucose and TAG inputs from the diet, and the functions $f_1(Y_L)$, $f_2(Y_L)$ and $f_3(Y_L)$ by

15 $$\begin{aligned} f_1(Y_L) = \displaystyle \frac{Y_L}{Y_0+Y_L} , \quad f_2(Y_L) = \displaystyle \frac{Y_\mathrm{max} - Y_L}{Y_0 + Y_\mathrm{max} - Y_L} , \quad f_3(T_L) = \displaystyle \frac{T_L}{T_0+T_L} . \end{aligned}$$

Note that the model given in Eqs. (1)–(15) is a generalisation of the one derived in Pearson et al. (2014), in that additional parameters $\sigma _Y,\sigma _L,\sigma _T,\sigma _A,\sigma _G,\phi _A$ have been introduced to model insulin sensitivity. The derivation of values for these parameters is discussed in the next section. Their values are summarised in Table 3, whilst Tables 1 and 2 contain a summary of the variables used in the model and a detailed list of parameters, respectively.

**Table 3:** Dimensionless insulin sensitivity (resistance) parameters, together with their values and references from which their values are inferred

Parameter	Description	Value	References
$\sigma _Y$	Hepatic glucose uptake sensitivity	0.5	Krssak et al. (2004)
$\sigma _L$	Hepatic glucose output sensitivity	0.06	Krssak et al. (2004)
$\sigma _T$	Hepatic TAG output sensitivity	0.25	Adiels et al. (2007)
$\sigma _A = \sigma _{AT}$	Adipose tissue FFA output & TAG uptake sensitivity	0.1	Bickerton et al. (2008)
$\phi _A = \phi _{AT}$	Adipose tissue FFA output & TAG uptake sensitivity	0.4	Bickerton et al. (2008)
$\sigma _G$	Skeletal muscle glucose uptake sensitivity	0.2	Basu et al. (2007)

Details of this procedure are given in “Appendix 1”

## Numerical Results

In this section, we use the model to explore the effects of insulin resistance on the various tissues of the body and the resultant effects on the other tissues. We produce results for the case of each individual tissue being insulin resistant, and the case where all tissues are resistant, and compare these results against simulations of the control case where no tissues are resistant. All simulations correspond to initial conditions of an overnight fast followed by ingestion of a healthy balanced meal containing 550 kcal of carbohydrates (glucose) and 150 kcal of fats (TAG). For the numerical solution, we use a quadratic approximation to the insulin production term, $f_0(G_b) = k_{IG} G_b + k_{I2} G^2_b$. The equations are solved in their non-dimensional form, all concentrations having been scaled by their postprandial steady-state values, so that all the corresponding non-dimensional concentrations are unity. This state is used as the initial conditions for a numerical solution of the system.

**Fig. 2:**
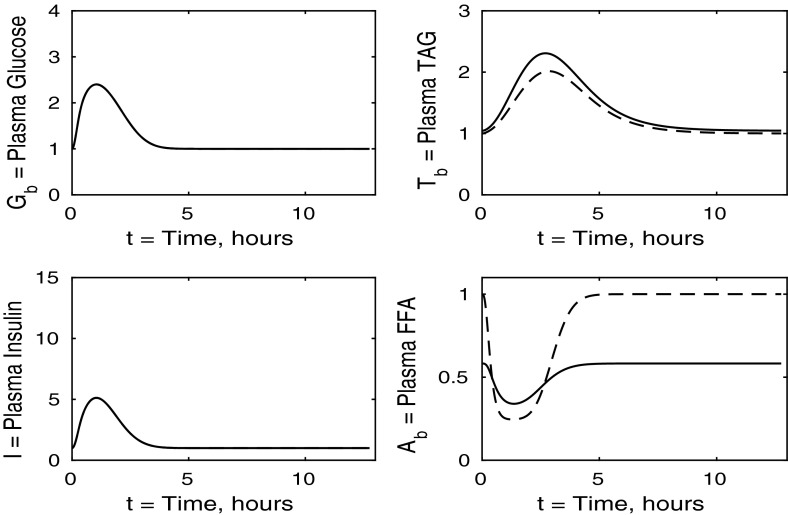
Postprandial kinetics for plasma glucose (*top left*), plasma insulin (*bottom left*), plasma TAG (*top right*) and plasma FFA (*bottom right*) for a balanced meal; the response of a healthy subject is indicated by the *dashed line* and the case of insulin-resistant adipose tissue by the *solid lines*

**Fig. 3:**
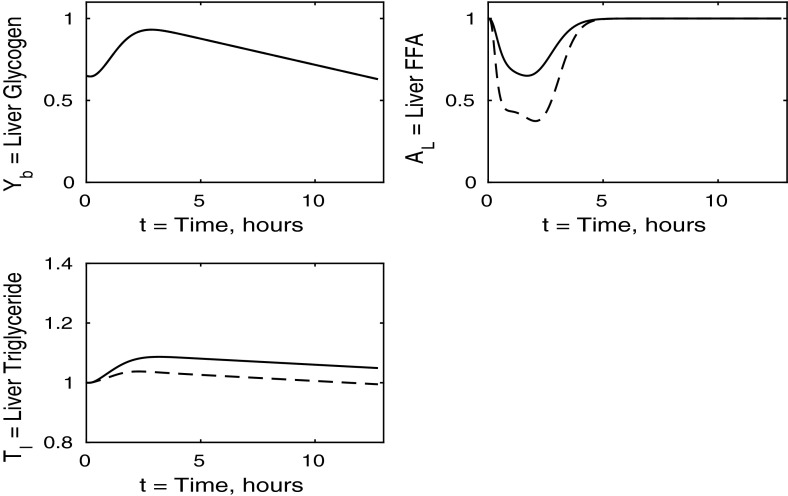
Postprandial kinetics for hepatic glycogen (*top left*), hepatic FFA (*top right*), hepatic TAG (*bottom*) for a balanced meal (14); the response of a healthy subject is shown by the *dashed lines* and the case of insulin-resistant adipose tissue by the *solid*

**Fig. 4:**
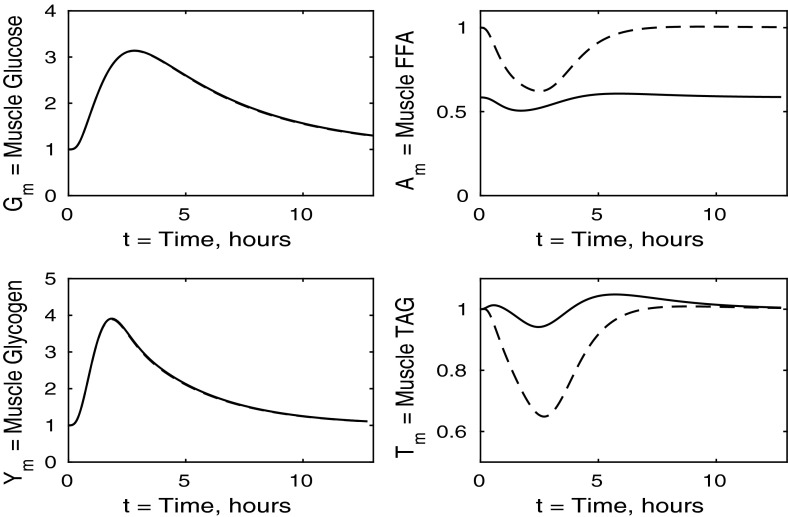
Postprandial kinetics for muscle glucose (*top left*), muscle glycogen (*bottom left*), muscle FFA (*top right*) and muscle TAG (*bottom right*) for a balanced meal. The *dashed line* shows the response of a healthy subject and the *solid line* the response of insulin-resistant adipose tissue

**Fig. 5:**
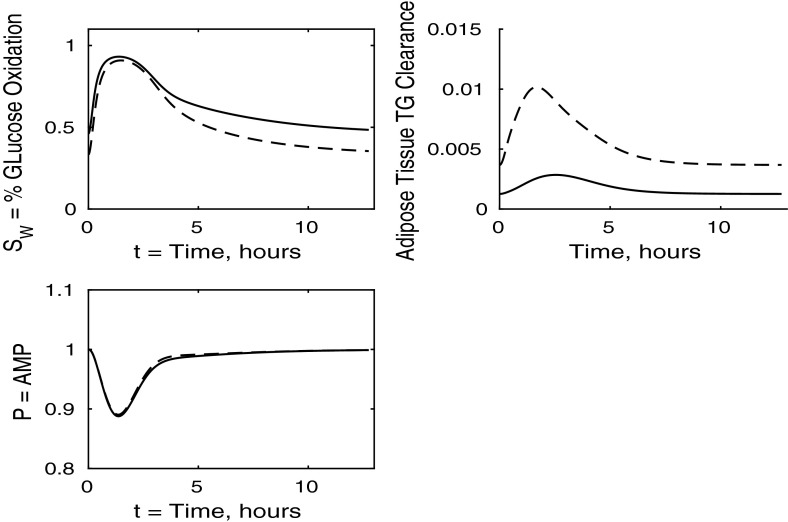
Postprandial kinetics for fractional glucose oxidation (*top left*), adipose tissue TAG clearance (*top right*) and *P*, our AMP marker (*bottom left*), for a balanced meal. The response of a healthy subject is shown by a *dashed line* and that of insulin-resistant adipose tissue by a *solid line*

The control case of no insulin resistance was studied in Pearson et al. (2014). The results for this case are illustrated by the dashed lines in Figs. 2, 3, 4 and 5. Figure 2 shows that the ingestion of the meal causes a spike in plasma glucose which, in turn, causes an increase in insulin, a reduction in plasma FFA and an increase in plasma TAG over a longer timescale. These increases in glucose and TAG are taken up by the liver and muscle, as shown in Figs. 3 and 4, where liver glycogen and TAG increases; similar behaviour is seen in muscle glucose and glycogen. The reduction in FFA is also seen in the liver and muscle, with muscle TAG also experiencing a reduction, due to the interconversion between FFA and TAG in the muscle. Figure 5 shows an increase in adipose tissue TAG clearance and reduction in AMP as the muscle switches to a FFA-glucose balance of energy usage to more heavily rely on the available glucose.

In the following sections, we consider the cases where each individual tissue is insulin resistant whilst the rest of the body has normal sensitivity. Finally, in Sect. 3.4, we discuss the case where all tissues are insulin resistant. For completeness, the cases in which two of the three tissues are insulin resistant whilst the third has normal sensitivity is discussed in “Appendix 2”. Whilst we acknowledge that such cases may not be as relevant physiologically, we include them for the sake of presenting a full analysis of the mathematical model.

### The Case Where Only Adipose Tissue is Insulin Resistant

We first discuss the results for plasma concentrations when only adipose tissue is insulin resistant, which are shown in Fig. 2. In this case we solve the system (1)–(15) with all the $\sigma _X$ ($X=A,AT,G,L,T,Y$) parameters set to unity, except for $\sigma _A=\sigma _{AT}=0.1$ and $\phi _A=\phi _{AT}=0.4$ as given in Table 3. The results for plasma glucose and insulin are indistinguishable to those for a healthy individual with no tissues insulin resistant. However, the results for plasma TAG and FFA show some differences. The baseline level of plasma FFA is lower in the insulin-resistant case. Following ingestion of a meal, plasma FFA levels still decrease for insulin-resistant adipose tissue, but by a much smaller amount than in the control. Also, plasma TAG is seen to rise to a slightly higher level following the meal.

We now turn to the liver concentrations shown in Fig. 3. These results show that hepatic glycogen storage is almost indistinguishable from that of a healthy subject. However, hepatic FFA levels are higher in individuals with insulin-resistant adipose tissue, which has the effect of increasing hepatic TAG content as well as leading to a net gain in hepatic TAG content. This increase in hepatic FFA, and subsequently TAG, is due to the increased plasma FFA levels following the meal caused by insulin resistance in the pathway for adipose tissue FFA output.

In Fig. 4 we show the muscle concentrations of glucose, glycogen, FFA and TAG. Following the meal, muscle glucose and glycogen levels are indistinguishable from healthy subjects (Fig. 4, upper and lower left panels). As with plasma, the fasting level of FFA in muscle is lower in the insulin-resistant case than in the healthy. The muscle FFA and TAG response to the meal, however, show that, although there is still an initial decline in muscle concentrations, it is less than in healthy individuals, and subjects with insulin-resistant adipose tissue subsequently exceed their steady-state values during the recovery phase, showing that there is an increased uptake of fat into skeletal muscle when adipose tissue is insulin resistant.

The results for our measure of metabolic flexibility, namely fractional glucose oxidation, together with *P*, our proxy for AMP concentration in muscle, and adipose tissue TAG clearance are shown in Fig. 5. These results show a slightly reduced metabolic flexibility when compared to those for a healthy individual, and adipose TAG clearance is reduced significantly in subjects with insulin-resistant adipose tissue. This clarifies why following ingestion of a meal the predicted plasma TAG level is higher than that of healthy individuals.

### The Case Where Only the Liver is Insulin Resistant

**Fig. 6:**
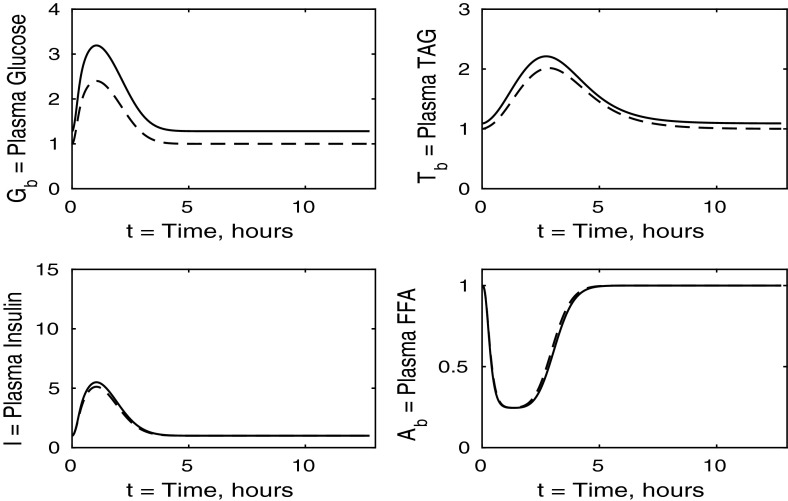
Postprandial kinetics for plasma glucose (*top left*), plasma insulin (*bottom left*), plasma TAG (*top right*) and plasma FFA (*bottom right*) for a balanced meal; the *dashed line* shows the response of a healthy subject and the *solid line* the case where the liver is insulin resistant

For this case, we take $\sigma _G = \sigma _Y = 1$ and $\phi _A = \phi _{AT} = 1$ and set $\sigma _L$, $\sigma _Y$ and $\sigma _T$ to the values given in Table 3. We discuss the results in the same order as in the previous section, first considering the plasma concentrations shown in Fig. 6. The insulin-resistant liver is broadly similar to that of a healthy individual; however, there is a slight increase in plasma glucose and insulin levels due to the liver not taking up as much glucose.

**Fig. 7:**
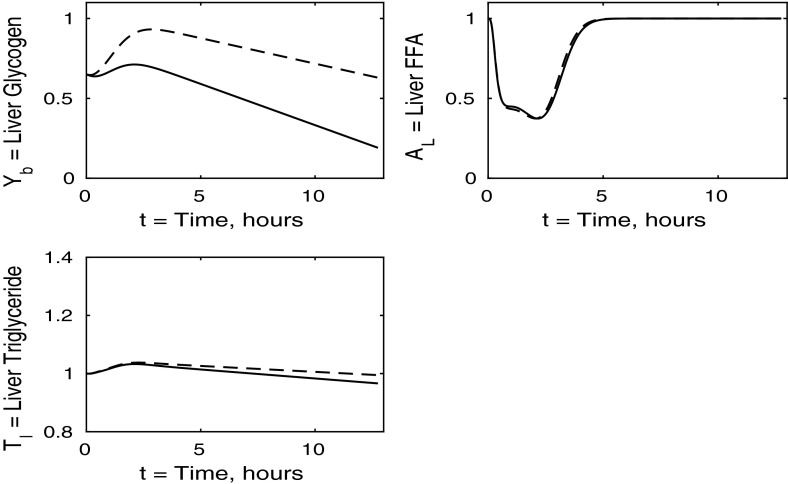
Postprandial kinetics for hepatic glycogen (*top left*), hepatic FFA (*top right*), hepatic TAG (*bottom*) for a balanced meal; the response of a healthy subject is shown by the *dashed line* and that of an insulin-resistant liver by a *solid line*

**Fig. 8:**
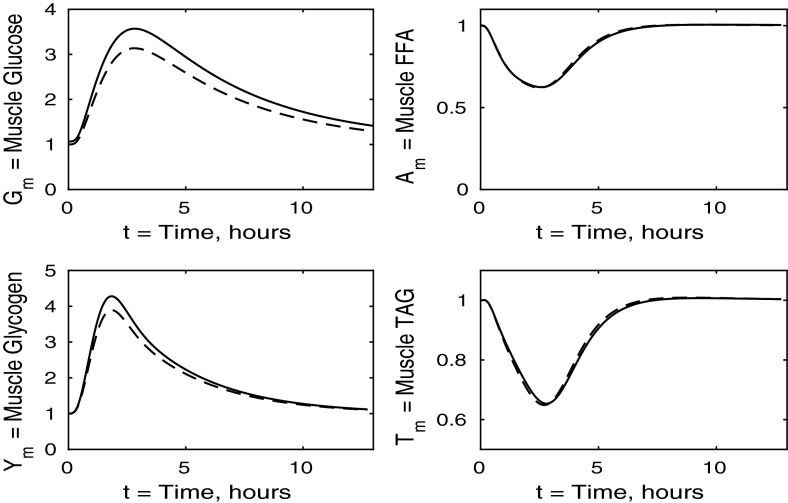
Postprandial kinetics for muscle glucose (*top left*), muscle glycogen (*bottom left*), muscle FFA (*top right*) and muscle TAG (*bottom right*) for a balanced meal; the response of a healthy subject is shown by the *dashed line* and that of an insulin-resistant liver by the *solid line*

**Fig. 9:**
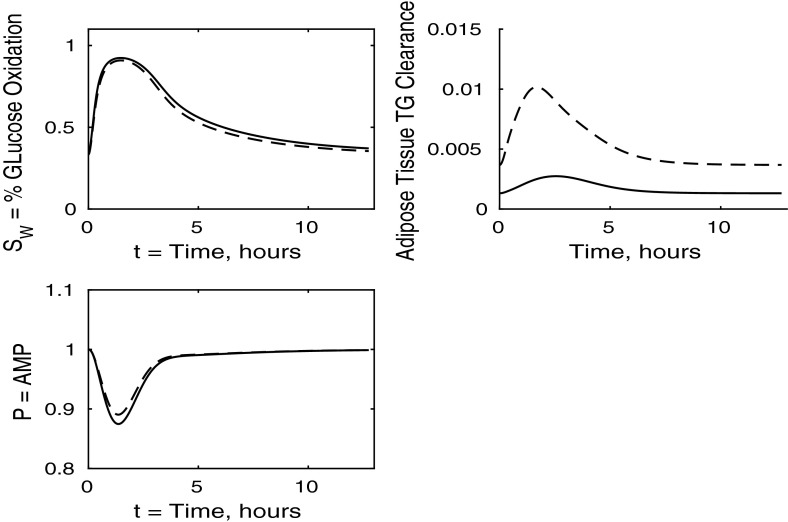
Postprandial kinetics for factional glucose oxidation (*top left*), adipose tissue TAG clearance (*top right*) and *P*, our AMP marker (*lower left*), for a balanced meal; the response of a healthy subject is shown by the *dashed line* and that of an insulin-resistant liver by a *solid line*

The liver concentrations are shown in Fig. 7. Hepatic FFA levels are similar to those of a healthy individual due to the transport between plasma and the liver of FFA being independent of insulin. Following the meal less glycogen and TAG are stored in the liver which result in a net reduction of both in the liver. From Figs. 8 and 9 this, instead, goes into muscle glucose, and glycogen, and adipose tissue, respectively. In the insulin-resistant liver, the hepatic concentration of glycogen is significantly lower than that for a healthy individual.

Figure 8 shows the muscle concentrations of glucose, glycogen, FFA and TAG when the liver is insulin resistant. We observe that muscle TAG and FFA are not affected by hepatic insulin resistance. Following ingestion of the meal, muscle glucose and glycogen levels rise to a slightly higher level than those of a healthy individual due to an increased muscle glucose uptake which partly compensates for the inability of the insulin-resistant liver to take up plasma glucose. Increased muscle glucose causes a slightly larger reduction in the muscle concentration of *P* shown in Fig. 9, and there is no significant difference in the fraction of oxidation due to glucose.

### The Case Where Only Skeletal Muscle is Insulin Resistant

**Fig. 10:**
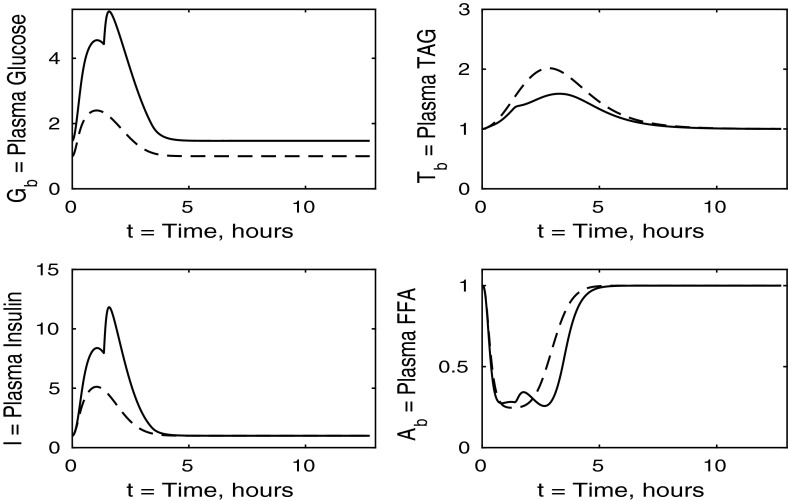
Postprandial kinetics for plasma glucose (*top left*), plasma insulin (*bottom left*), plasma TAG (*top right*) and plasma FFA (*lower right*) for a balanced meal; the response of a healthy subject is shown by the *dashed line* and that of insulin-resistant skeletal muscle by a *solid line*

The case of insulin-resistant skeletal muscle produces the most interesting results. We set all $\sigma _X$ and $\phi _Z$ variables to unity ($X=AT,G,L,T,Y, Z=A,AT$), with the sole exception of $\sigma _G$, which is set at 0.15, as given in Table 3 and derived in “Skeletal Muscle Glucose Uptake” section in “Appendix 1”. Again, we start with the plasma concentrations, which are presented in Fig. 10. We clearly see that postprandial glucose and insulin levels are higher than in healthy individuals and they also exhibit a strange double spike which affects other concentrations as insulin is the main regulatory hormone for many pathways in our model. This double spike is noticeable because we have plotted the solutions of our differential equations as continuous curves, and if a sequence of points had been shown, it would not be noticeable. The reason for the double spike is the liver glycogen store having a fixed maximum capacity, which suddenly alters the nature of the equations when it is filled; in reality this effect would be smoothed out. The raised insulin levels cause a reduction in plasma TAG and an extended period of reduced FFA concentrations.

**Fig. 11:**
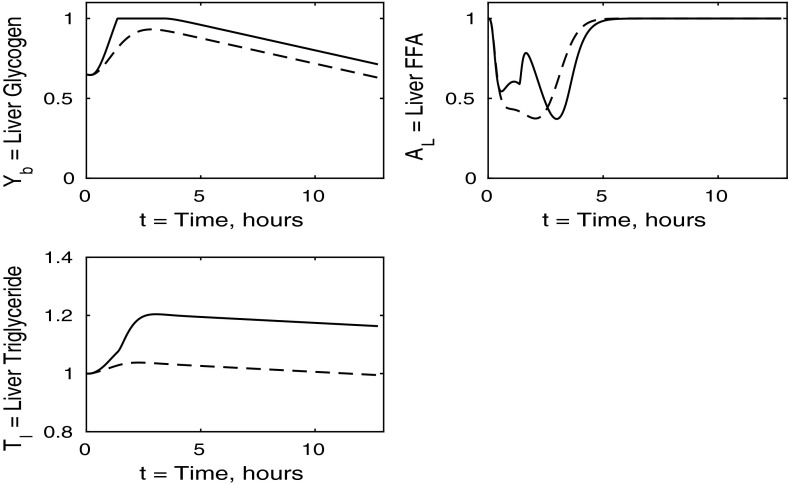
Postprandial kinetics for hepatic glycogen (*top left*), hepatic FFA (*top right*), hepatic TAG (*bottom*) for a balanced meal; the response of a healthy subject is indicated by the *dashed line* and that of insulin-resistant skeletal muscle by a *solid line*

The liver concentrations shown in Fig. 11 explain the unusual results in the blood plasma. Following ingestion of the meal the liver becomes saturated in glycogen and is unable to clear excess glucose from the plasma. This leads to the second spike in plasma glucose and insulin. We observe a net gain in liver TAG content caused by an increase in hepatic FFA at earlier times, of $0<t<3$, which corresponds to 3 h.

**Fig. 12:**
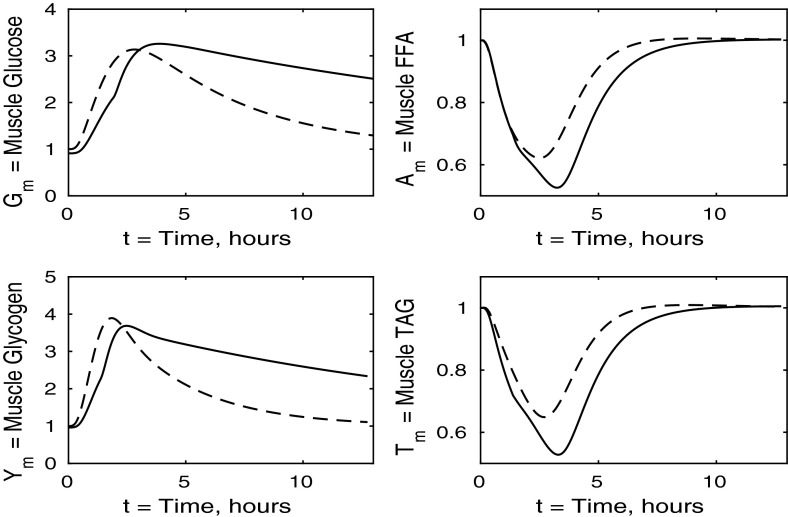
Postprandial kinetics for muscle glucose (*top left*), muscle glycogen (*bottom left*), muscle FFA (*top right*) and muscle TAG (*bottom right*) for a balanced meal; the *dashed lines* show the response of a healthy subject and the *solid lines*, the case of insulin-resistant skeletal muscle

Turning to Fig. 12, we note that muscle FFA and TAG concentrations drop to lower levels, before recovering their steady- state values, this occurring on a similar timescale to that for normal subjects. Thus in the muscle, insulin resistance has the major effect on carbohydrate processes and only a minor effect on mechanisms involving fats.

**Fig. 13:**
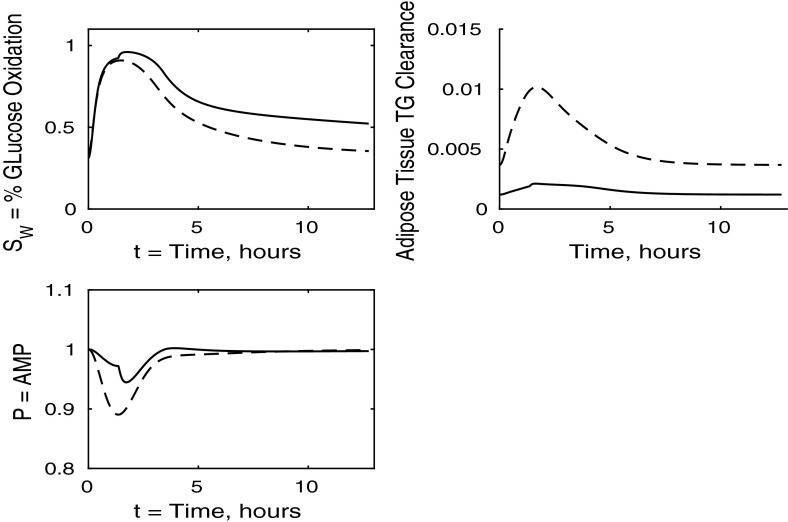
Postprandial kinetics for fractional glucose oxidation (*top left*), adipose tissue TAG clearance (*top right*), *P* our AMP marker (*lower left*) for a balanced meal. The *dashed lines* show the response of a healthy subject, and the case of insulin-resistant skeletal muscle is shown by a *solid line*

The percentage of glucose oxidation, the muscle concentration of *P* and the adipose TAG clearance are shown in Fig. 13. From Fig. 12 we note that muscle glucose and glycogen levels rise to similar values as in healthy individuals, despite reduced plasma glucose uptake. However, both glucose and glycogen take much longer to return to steady-state levels. This is due to the lower rate of glucose oxidation occurring in the muscle. The results initially show a lower percentage of glucose oxidation than in healthy individuals; however, this ratio still reaches a similar peak postprandially due to insulin levels being much higher in the insulin-resistant muscle and the amount of FFA available for oxidation in the muscle also being reduced. This explanation is supported by our results for *P*, which imply less total oxidation occurring in the muscle. The graph of fractional glucose oxidation surprisingly shows that the metabolic flexibility; that is, the range of fractional oxidation is smaller for the IR cases than the healthy. Our results show a decrease in adipose TAG clearance over that for healthy individuals.

### The Case Where All Tissues Are Insulin Resistant

**Fig. 14:**
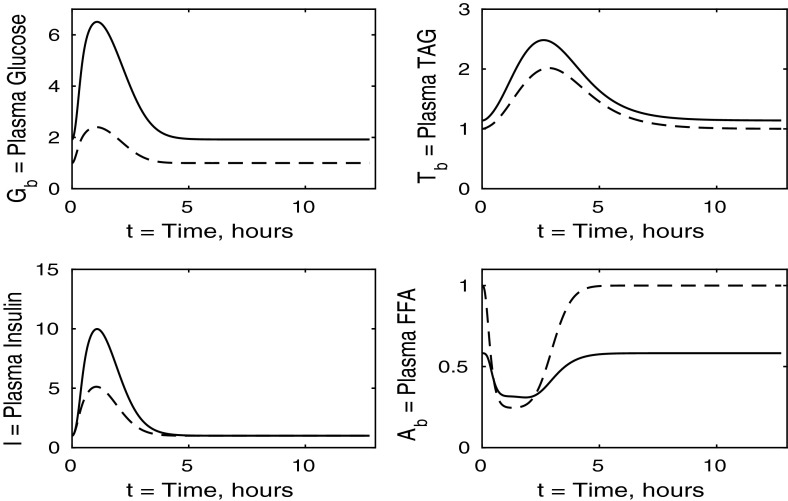
Postprandial kinetics for plasma glucose (*top left*), plasma insulin (*bottom left*), plasma TAG (*top right*) and plasma FFA (*bottom right*) for a balanced meal. The *dashed line* shows the response of a healthy subject and the *solid line* the case where all tissues are insulin resistant

We now solve the model with all tissues insulin resistant, that is, when all $\sigma _X$ and $\phi _Z$ parameters ($X=A,AT,L,G,Y,T$, $Z=A,AT$) take the values given in Table 3. We first describe the results for plasma concentrations shown in Fig. 14. Following the meal, plasma glucose, TAG and insulin all rise to higher levels than in a healthy individual, whilst plasma FFA is suppressed to a lesser extent. The plasma levels in this simulation are higher than when any one tissue is insulin resistant. This increased effect is caused by a greater total reduction in plasma glucose clearance due to every tissue being insulin resistant.

From Fig. 14 we observe that in the insulin-resistant case, the fasting level of plasma TAG is higher than in the healthy state. This is typically expected and supports the suggestion of McLaughlin et al. (2003) that fasting plasma TAG could be used as an indicator of insulin resistance. We note that this is not the case when only muscle is resistant (Fig. 10), is a small effect when adipose tissue is resistant (Fig. 2), and a slightly larger effect when liver is resistant (Fig. 6).

**Fig. 15:**
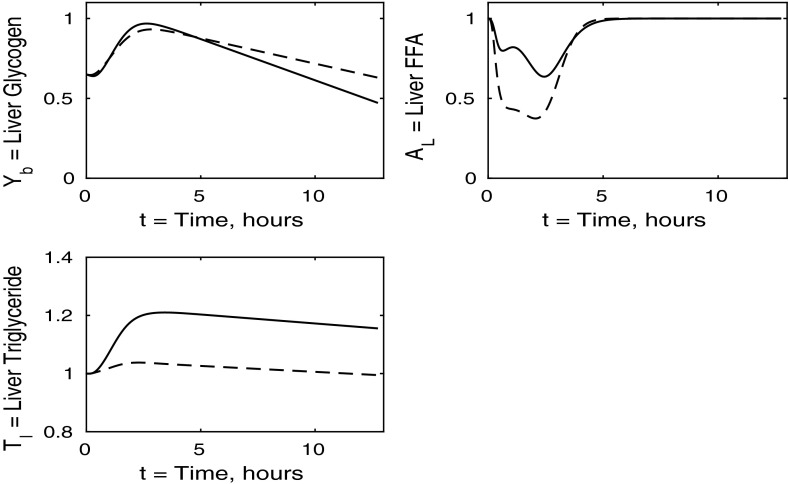
Postprandial kinetics for hepatic glycogen (*top left*), hepatic FFA (*top right*), hepatic TAG (*bottom*) for a balanced meal. The results for a healthy subject are indicated by the *dashed lines*, and the *solid line* shows the case where all tissues are insulin resistant

The liver concentrations are shown in Fig. 15. Hepatic glycogen behaves similarly in the healthy individual to the case where all tissues are insulin resistant. This is due to the plasma glucose and insulin levels being higher in the insulin-resistant case, which counteracts the reductions in uptake and release rates caused by the insulin resistance, and leads to similar fluxes. Following the meal, hepatic FFA shows a much smaller suppression in the case where all tissues are insulin resistant. However, hepatic TAG exhibits a significant net gain following the meal which, as we shall see below, is due to insulin resistance suppressing TAG take-up by adipose tissue, leaving plasma TAG levels elevated for an extended period of time.

**Fig. 16:**
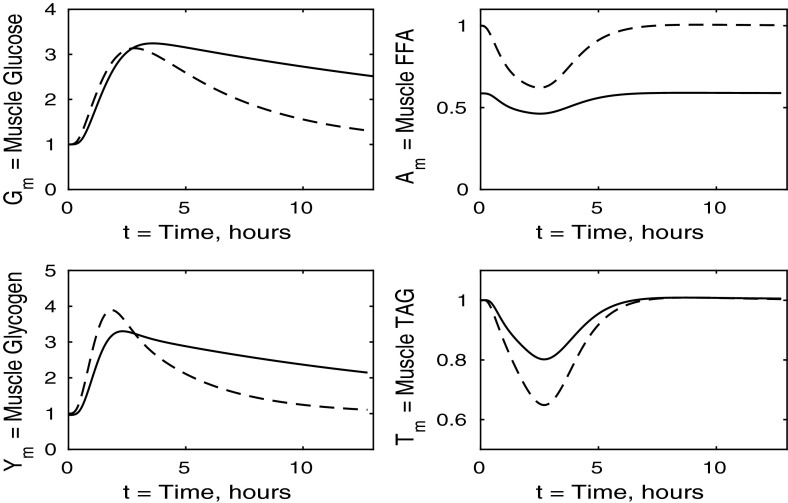
Postprandial kinetics for muscle glucose (*top left*), muscle glycogen (*bottom left*), muscle FFA (*top right*) and muscle TAG (*bottom right*) for a balanced meal. The response of a healthy subject is shown by the *dashed line*, and the *solid lines* indicate the case where all tissues are insulin resistant

Figure 16 shows that muscle glucose and glycogen rise to similar levels as in healthy subjects, due to the increased plasma glucose; however, they take much longer to return to their steady states compared to healthy individuals. This slow behaviour is due to glucose oxidation being insulin stimulated. These results when all tissues are insulin resistant are similar to those for the case where only muscle is insulin resistant, and again, insulin resistance lowers the fasting levels of FFA in the muscle tissue. The timescales for muscle TAG and FFA equilibrium are similar to those for healthy individuals, although we see a smaller suppression following the meal, due to the increased postprandial plasma FFA being taken up into the muscle tissue independently of insulin. The timescale over which muscle FFA and muscle TAG return to steady state is unaffected by insulin resistance.

**Fig. 17:**
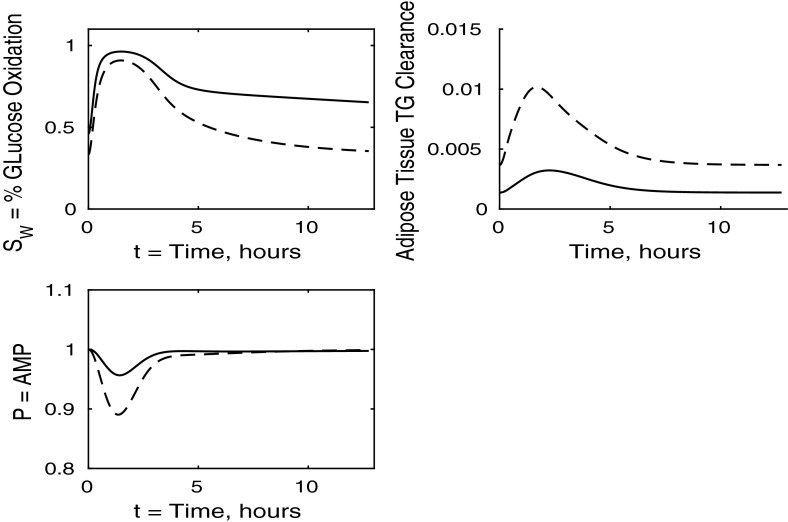
Postprandial kinetics for factional glucose oxidation (*top left*), adipose tissue TAG clearance (*top right*) and *P* our AMP marker (*lower left*) for a balanced meal. The *dashed lines* show the response of a healthy subject, and *solid lines* correspond to the case where all tissues are insulin resistant

Figure 17 shows the fraction ratio of oxidation due to glucose together with the muscle concentration *P* (our marker for AMP) and adipose TAG clearance. In the case of whole-body insulin resistance, there is less total oxidation and a smaller fraction of glucose oxidation following the meal. Thus, insulin resistance causes reductions in metabolic flexibility and in the total oxidation occurring, leading to an accumulation of unused substrates in the muscle. The end result of this is a significant increase in the time taken for the muscle to return to the fasting state; in fact, our simulations show that even after 12 h, the fasting state has not been recovered. There is also a significant *reduction* in the amount TAG cleared from the plasma to adipose tissue, which compensates for the increased TAG levels in the plasma and liver; this may lead to non-alcoholic fatty liver disease.

### Discussion

Comparing the results of all tissues being insulin resistant with the cases of just one individual tissue, we can make several inferences. First, we note that left panels in Fig. 14 have greater similarity with Fig. 10 than Figs. 2 or 6, suggesting that the changes in plasma glucose and insulin are determined mainly by the insulin resistance of the muscle rather than any insensitivity in the adipose or hepatic tissue. In contrast, changes in the plasma FFA is more due to adipose tissue insensitivity rather than liver or muscle. That plasma TAG shows little change between normal and all tissues being insulin resistant is due to some cancellation between the effects of insulin resistance in the muscle and adipose tissue.

There is some cancellation of the effects of resistance of muscle and liver in liver glycogen; see the top left panels of Figs. 7, 11 and 15. The lower left panels suggest that the change in liver TAG is partly due to insulin resistance of the muscle, and change in liver FFA is a combination of insulin resistance of adipose tissue and muscle as well as the liver itself, and not just due to the liver being insulin resistant. The development of a fatty liver is a complex process, and there will be other effects also, for example the insulin-dependent activity of SREBP1c and Akt2 as noted by Shimomura et al. (2000) and Wan et al. (2011).

All panels of Fig. 16 show similar effects to Fig. 12 (insulin-resistant muscle), with some influence of adipose insensitivity on muscle FFA and muscle TAG (the exception being the lower fasting concentration of muscle FFA caused by the insulin resistance of adipose tissue). Similar comments can be made with regard to Figs. 17 and 13, where the effects of insulin resistance on *P* and relative glucose oxidation are primarily due to muscle insensitivity rather than hepatic or adipose tissue, although insulin resistance of adipose tissue has some influence of adipose TAG clearance.

## Conclusions

In this paper, we extended a mathematical model of human metabolism, described in detail in an earlier paper, in order to test the effects of insulin resistance on individual tissues in the body. The motivation for this is to gain insight into the development of diabetes and provide insight into the answers to questions such as “does insulin resistance start in one tissue and spread to the others?” and “which tissue develops insulin resistance first?”. We referred to the literature to parametrise our model for insulin resistance and took account of the fact that tissues are affected by insulin resistance to different degrees.

Our results show that when one tissue in the body is insulin resistant, reduced metabolism causes increased levels of glucose and fats in plasma which affects other tissues in the body. The largest effect is the liver becoming overloaded with glucose and rapidly reaching its maximum storage capacity when skeletal muscle is insulin resistant. This leads to a double spike in plasma glucose, which causes the same behaviour for plasma insulin, and affects the other components. However, in the situation where *all* tissues are insulin resistant, we do not see the same results, as the liver glycogen does not reach its maximum capacity, as its ability to take up glucose is itself impaired by insulin resistance.

There are two plausible conclusions that can be drawn from this. The first is that when the skeletal muscle is insulin resistant, the liver is overloaded, and over time, this causes the liver to stop functioning normally and develop insulin resistance. Another possibility is that, because the results for insulin-resistant skeletal muscle have a strange double peak, they are unphysiological, and skeletal muscle is never, or rarely, the first tissue to develop insulin resistance. However, the strange form is due to the model having a sharp switch off of glucose transport into the liver when the liver’s glycogen store reaches its maximum level. In reality, this switch off would be smoothed out by intermediate processes, and the minimum between the two maxima (which in simulations is quite shallow and only lasts for a few minutes) would not be observed in experiments. There is significant evidence that muscle is the first tissue to become insulin resistant; see, for instance, DeFronzo and Tripathy (2009). However, even if insulin resistance in one tissue causes resistance in another, the development of insulin resistance in both tissues will be a gradual process. One would not expect to simultaneously find the insulin sensitively of muscle severely compromised whilst liver and adipose tissues were healthy. Tissues do not suddenly switch from normal sensitivity, $\sigma = 1$, to resistant $\sigma =0.5$ or 0.2 (see Table 3 for sensitivities used here). Instead, there will be a gradual reduction in $\sigma$ values in all tissues concerned.

A further conclusion we can draw from our results is the effect of insulin resistance on the fat content of the liver. It is still currently unknown whether a fatty liver is the cause or an effect of insulin resistance. Our results have indicated that insulin resistance can cause a net gain in liver fat, and this can occur when tissues *other* than the liver were insulin resistant.

As described above, it seems that when one tissue is insulin resistant it causes the others to become over-worked in order to compensate, which could lead to insulin resistance also developing in these other tissues. Although hepatic insulin resistance does not increase hepatic fat, once insulin resistance is developed in the whole body, we see a rise in hepatic fat. Therefore, our results support the hypothesis that a fatty liver is caused by insulin resistance; however, we are unable to test the converse with our model, namely that fatty liver leads to insulin resistance.

We are also interested in the metabolic flexibility in skeletal muscle. Our results show that when the liver or adipose tissue is insulin resistant there is little effect on the percentage of oxidation due to glucose or on the total oxidation occurring. However, in the case where skeletal muscle is insulin resistant there is a significant decrease in metabolic flexibility (percentage of glucose oxidation), as well as in both the preprandial percentage of glucose oxidation and the total amount of oxidation both being reduced. These effects are seen to a greater extent when the whole body is insulin resistant despite the fact that when only liver or adipose tissue was insulin resistant it had only a slight effect.

Our results suggest that the liver develops insulin resistance earlier than muscle and adipose tissue. This is seen from the increase in liver fat early in the process and is consistent with observations of fatty liver in subjects of normal weight. Our analysis which assumes one tissue is resistant whilst others have normal insulin sensitivity is an oversimplification. In reality insulin resistance arises as a progressive deterioration. Thus in the early stages, one would expect a small increase in fasting insulin, then a small increase in fasting glucose and a larger increase in insulin and eventually a rise in fasting triglycerides. Eventually, of course insulin release cannot keep up with the resistance and insulin levels fall, glucose rises and diabetes occurs. To model the early stages of IR, we have taken the increases in fasting insulin and glucose to be negligibly small.

As the current model is focused on the response to a meal, one factor missing is the effect on the fasting glucose level, which is raised in diabetes and prediabetes. The reason for this is commonly thought to be due to hepatic insulin resistance; see, for example, Bock et al. (2007). This effect could be accounted for in the model by incorporating the glucose sensitivity of the pancreas in the insulin production terms in Eq. (1) and in reducing the rate at which insulin is broken down. The current work Pratt et al. (2015a), Pratt et al. (2015b), Pratt (2015) focuses on improving the model by expanding it to give a more complete model of human metabolism by including more intermediate stages the metabolic pathways, more transport terms (e.g. for pyruvate), a more accurate description of insulin production, metabolism in adipose tissue and, in addition to glucose, the input of fructose representing a non-insulin stimulating carbohydrate.

## Figures and Tables

**Fig. 18:**
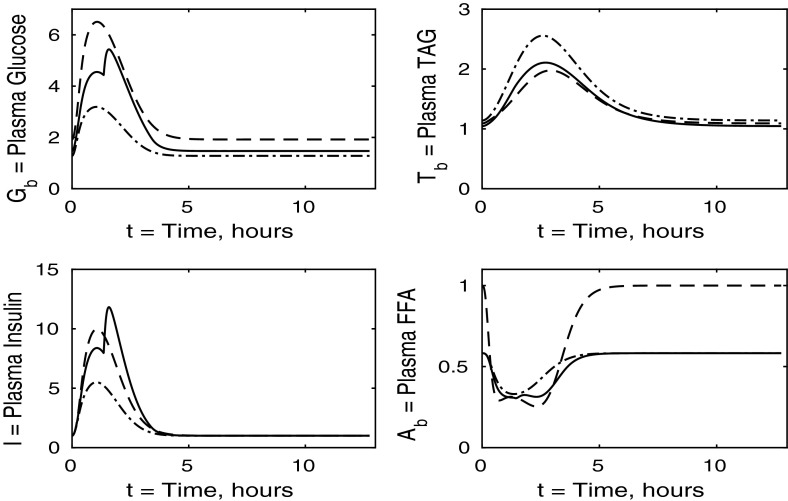
Postprandial kinetics for plasma glucose (*top left*), plasma insulin (*bottom left*), plasma FFA (*top right*) and plasma TAG (*bottom right*) for a balanced meal, with adipose and muscle insulin resistance (*solid line*), hepatic and muscle insulin resistance (*dashed line*), and adipose and liver insulin resistance (*dash-dotted line*)

**Fig. 19:**
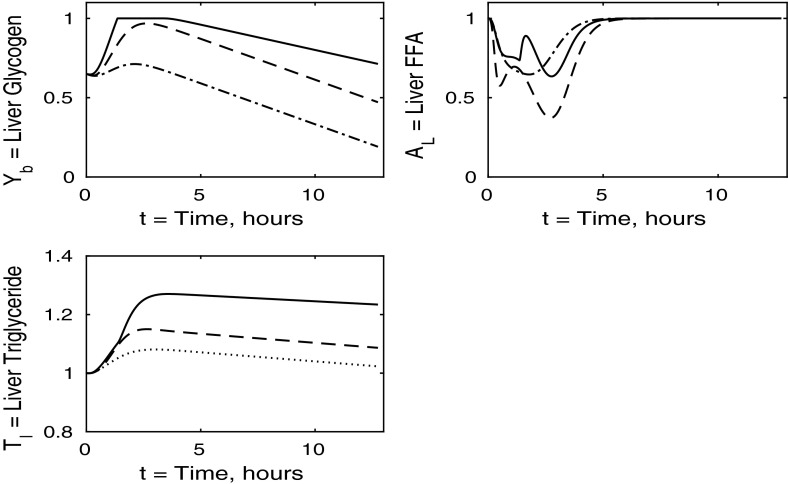
Postprandial kinetics for liver glycogen (*top left*), liver TAG (*bottom*) and liver FFA (*top right*) for a balanced meal, with adipose and muscle insulin resistance (*solid line*), hepatic and muscle insulin resistance (*dashed line*), and adipose and liver insulin resistance (*dash-dotted line*)

**Fig. 20:**
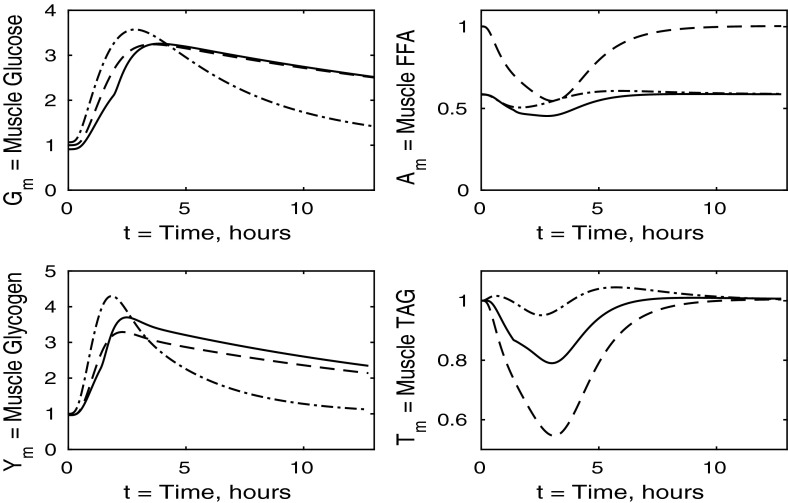
Postprandial kinetics for muscle glucose (*top left*), muscle glycogen (*bottom left*), muscle FFA (*top right*) and muscle TAG (*bottom right*) for a balanced meal, with adipose and muscle insulin resistance (*solid line*), hepatic and muscle insulin resistance (*dashed line*), and adipose and liver insulin resistance (*dash-dotted line*)

**Fig. 21:**
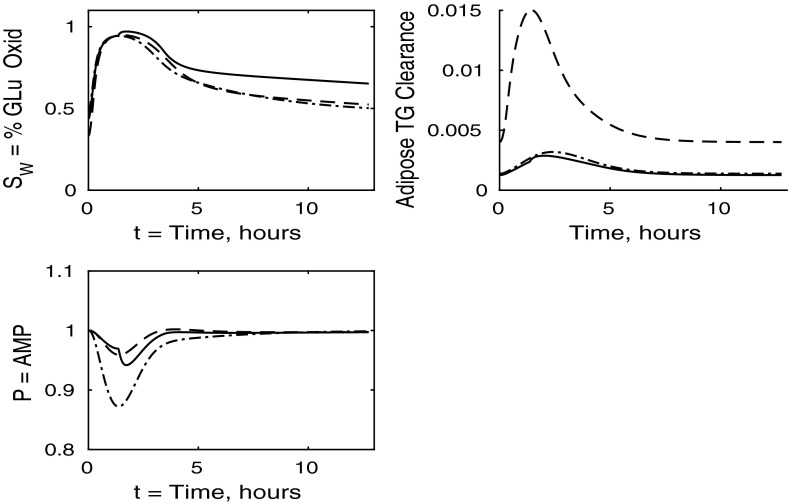
Postprandial kinetics for fractional glucose oxidation (*upper left*), adipose tissue TAG uptake (*upper right*) and muscle P, our marker of AMP (*lower left*), following a balanced meal. Key: adipose and muscle insulin resistance (*solid line*), hepatic and muscle insulin resistance (*dashed line*) and adipose and liver insulin resistance (*dash-dotted line*)

## Appendix 1: Parametrising Insulin Resistance

In this appendix, we describe how we parametrise our model to describe the insulin resistance in each of the tissues. This is achieved through the $\sigma _X$ parameters ($X=A,AT,G,L,T,Y$), which are more accurately described as insulin sensitivities. We take $\sigma _X=1$ to represent normal sensitivity, and $\sigma _X < 1$ to indicate reduced sensitivity, which is a measure of insulin resistance.

### Hepatic Lipogenesis

Peterson et al. (2007) studied the effect of skeletal muscle insulin resistance on metabolic syndrome. The study took a group of insulin-resistant subjects and a group of insulin-sensitive subjects who, following an overnight fast, were fed two test meals. Measurements were taken of liver and muscle glycogen and lipids. Of particular interest to us are the data comparing hepatic lipogenesis in insulin-resistant and insulin-sensitive subjects. The results showed that approximately twice the amount of lipogenesis occurred in the insulin-resistant subject compared to the insulin-sensitive. The results also showed that plasma insulin levels in the insulin-resistant subjects were approximately twice those of the insulin-sensitive. Hepatic lipogenesis is represented in our model by the term $k_{AL}IG_b/\alpha$ in (3) and (7); in our model the increase in hepatic lipogenesis can be entirely described by the difference in plasma insulin levels and no extra parameter describing insulin sensitivity on this pathway is needed.

### Hepatic Glucose Uptake and Hepatic Glucose Output

Krssak et al. (2004) have carried out a study into postprandial hepatic glycogen metabolism in type 2 diabetes after both a mixed meal and a hyperglycaemic hyperinsulinaemic clamp. Both sets of results are useful to us. During the clamp, the rate of hepatic glycogen storage in diabetic subjects was measured to be approximately 50 % that of the controls, with plasma insulin and glucose clamped at approximately the same concentration in both controls and diabetic subjects. The rate of hepatic glycogen storage is given in our model by the term $\sigma _Y k_L I G_b f_2(Y_L) / \eta$ in (3) and (6), where we have introduced the parameter $\sigma _Y$ to model insulin resistance. In order to achieve a 50 % reduced glycogen storage in insulin-resistant subjects we set $\sigma _Y=0.5$.

Of interest to us from the data for the mixed meal are the preprandial rates for endogenous glucose production. The results for the diabetic subjects also showed the production of approximately 2.0 mg/kg/min with a preprandial insulin level of approximately 11 pmol/l, compared to the control subjects which showed a glucose production rate of approximately 1.7 mg/kg/min with a preprandial insulin level of approximately 5 pmol/l. The term in Eqs. (3) and (6) of our model that describes this is given by $\beta _Gf_1(Y_L)/(\alpha (1+\sigma _Lk_{GL}I^2))$ where we have introduced the parameter $\sigma _L$ to model the insulin sensitivity involved with hepatic glucose output. We choose $\sigma _L=0.06$ to fit our model to the results.

### Hepatic TAG Output

Adiels et al. (2007) carried out a study into VLDL secretion rates. Their results showed that, during the clamp, insulin-resistant subjects had an approximately 10 % suppression of hepatic TAG output compared to a 50 % suppression for the control subjects. At the same time, the plasma insulin levels during the clamp were measured at 600 pmol/L for the insulin resistant and 500 pmol/L for the controls and before the clamp at 100 pmol/L for insulin resistant and 50 pmol/L for the controls. We denote our parameter for hepatic TAG insulin sensitivity by $\sigma _T$, and the term in our model describing hepatic TAG output is $\beta _T/(\alpha (1+ \sigma _T k_{TL} I))$ in Eqs. (4) and (8). If we choose $\sigma _T$ such that the hepatic TAG output during the clamp is 90 % of what it was before the clamp, we obtain a value of $\sigma _T \approx 0.25$.

### Adipose Tissue FFA Output and TAG Uptake

Bickerton et al. (2008) carried out a study into adipose tissue fatty acid metabolism in insulin-resistant and healthy subjects. The study commenced after an overnight fast followed by a meal of equal amounts of fat and carbohydrates (40 grams of each). Blood samples were taken before and for 6 h after the meal and rates of uptake into adipose tissue calculated. The first data of use to us from this paper are those for adipose tissue NEFA (FFA) output. Preprandial adipose FFA output was measured at approximately 600 nmol/min/100 g adipose tissue in the insulin-resistant subjects compared to approximately 1200 nmol/min/100 g tissue in the controls, whilst postprandial output was measured at approximately 200 nmol/min/100 g tissue in both the insulin-resistant and control subjects. Plasma insulin was measured at 50 pmol/L preprandial and 200 pmol/L postprandial for the controls and 100 pmol/L preprandial and 350 pmol/L postprandial for the insulin-resistant subjects.

To model the insulin sensitivity of FFA output from adipose tissue, we need to take account of two effects noted by Bickerton et al. (2008). First, there is decreased output of FFA from adipose tissue in the fasting state. Second, whilst raised insulin levels cause a decrease in FFA output, this decrease is reduced by insulin resistance. Thus, we introduce two insulin sensitivities $\sigma _A$ and $\phi _A$ and modify the output term from $\beta _A/(\alpha (1+k_{AA}I^2))$ to $\phi _A \beta _A /(\alpha (1+\sigma _A k_{AA}I^2))$. We set $\phi _A$ so that the preprandial adipose FFA output in insulin-resistant subjects is half that of the controls. This yields a value of $\phi _A \approx 0.4$. We set $\sigma _A$, which premultiplies $I^2$, to $\sigma _A \approx 0.1$, so that the change in plasma insulin for insulin-resistant subjects causes the output of FFA from the adipose tissue to be reduced to one-third of its preprandial level.

The paper also has useful data on adipose tissue TAG uptake. Over the 6-h postprandial period, the rate of uptake for the insulin-resistant subjects is approximately half that of the control subjects. We also note that the postprandial reduction in adipose TAG clearance is about 50 % in both the insulin-resistant and control subjects, although the insulin-resistant group have a higher plasma insulin concentration. If we take a similar approach to that adopted for adipose NEFA output and introduce two parameters to model insulin resistance then the term in our model for adipose TAG uptake in Eq. (4) is $\phi _{AT} k_{TA}(1 + \sigma _{AT}k_{AI}I)T_b$. Now if we set $\phi _{AT}=\phi _A=0.4$ and $\sigma _{AT}=\sigma _A=0.1$ then our model achieves results that correspond to the above experimental data.

### Skeletal Muscle Glucose Uptake

Basu et al. (2007) carried out a study of glucose uptake in healthy and diabetic subjects. They compared the muscle with splanchnic uptake, that is, by the visceral, or abdominal organs. The study started after an overnight fast, and all subjects were given a glucose infusion to maintain the arterial glucose concentration at approximately 9.3 mmol/l for both the control and diabetic subjects. All subjects were given a constant insulin infusion at two rates, an initial low insulin infusion and later a high insulin infusion. The resultant arterial insulin concentrations in the low insulin infusion subjects was approximately 80 pmol/l in the diabetic subjects and approximately 70 pmol/l in the controls; the high insulin infusion rates were approximately 170 pmol/l in the diabetics and approximately 140 pmol/l in the control subjects.

The results of particular interest to us are those for leg glucose uptake during the insulin infusions as we can take this as a guide for skeletal muscle glucose uptake. Leg glucose uptake, during the low insulin infusion, was measured in diabetic subjects to be three-fifths that of the controls (approximately 15 $\mu$mol/kg/min and approximately 25 $\mu$mol/kg/min, respectively), and during the high insulin infusion it was measured in diabetics to be two-fifths of that in the controls (approximately 20 $\upmu$mol/kg/min and approximately 50 $\mu$mol/kg/min, respectively, from Fig 4a of Basu et al. 2007). The term in our model which describes skeletal muscle glucose uptake is given by $k_G (1 + k_{GI}I \sigma _G)G_b/\alpha$, where the parameter $\sigma _G$ in Eqs. (3) and (10) describes the insulin sensitivity. We choose $\sigma _G$ such that the ratio between skeletal muscle glucose uptake in healthy ($\sigma _G=1$) and insulin-resistant simulations is the same as highlighted in the experimental data. Using the values from the data for the low insulin infusion gives a value of $\sigma _G \approx 0.2$. We choose the data for the low insulin infusion rate as this rate produces representative concentrations.

## Appendix 2: Analysis of Insulin Resistance of Pairs of Tissues

In this appendix, we show the results of our model when two tissues are insulin resistant. We thus consider the cases liver and adipose tissue resistant, adipose tissue and skeletal muscle resistant, and the liver and the muscle tissue resistant. As before we have simulated a healthy balanced meal ingested following an overnight fast and we start with the results for plasma concentrations shown in Fig. 18.

By comparing the results in Fig. 18 with those presented in Fig. 2, we see that no combination of two tissues being insulin resistant produces a large change in the results for plasma FFA and TAG. Also we see that an insulin-resistant liver and adipose tissue also have only a small effect on plasma glucose and insulin. Of particular interest are the results for an insulin-resistant liver and muscle where the combination of both tissues being resistant does not allow for the liver glycogen store to fill and, rather than producing the dual peak results that we saw in Sect. 3.3 (and here with resistant muscle and adipose tissue), we instead have the highest plasma glucose concentrations, with the knock-on effect of higher plasma insulin.

We now consider the results for the liver shown in Fig. 19 which show that when both the muscle and adipose tissue are insulin resistant then the hepatic glycogen store is again saturated due to the decrease in muscle glucose clearance from the blood. In both cases in which the liver is insulin resistant, there is a net loss of hepatic glycogen and TAG over the 12- to 14-h period although when the skeletal muscle is also resistant the increased plasma glucose levels result in slightly higher hepatic glycogen and TAG levels. The results for insulin-resistant liver and adipose tissue are very similar to those for where only the liver is insulin resistant.

Turning to the predicted concentrations in the muscle shown in Fig. 20, we observe that any combination of insulin resistance in tissues does not produce a large difference in the initial peaks of muscle glucose or glycogen. However, when the muscle is insulin resistant the timescale over which steady state is reestablished is longer due to a decrease in total muscle oxidation, as shown by the results for fractional glucose oxidation and *P*, as shown in Fig. 21. We also see that insulin resistance of adipose tissue results in lower levels of fasting muscle FFA and a suppression of the normal reduction of muscle TAG following a meal.
